# Psychiatric and behavioral problems in Prader-Willi syndrome: a clinical case

**DOI:** 10.1192/j.eurpsy.2025.1548

**Published:** 2025-08-26

**Authors:** I. M. Marques, S. Abreu, M. C. Coroa

**Affiliations:** 1 Department of Psychiatry, Centro Hospital e Universitário de Coimbra, Coimbra, Portugal

## Abstract

**Introduction:**

Prader-Willi syndrome (PWS) is a rare neurodevelopmental and multisystemic disorder. This syndrome is most often caused by paternal deletion or a maternal disomy of chromosome 15. PWS is characterized by hypotonia, hypogonadism, and hyperphagia. Intellectual disability, impaired social skills, emotional regulation, sleep disorders and behavioral problems (tantrums, temper outbursts, obsessive–compulsive symptoms, skin picking) are also present. Autism spectrum disorder, mood disorders, anxiety, and psychosis are common in these individuals. (Bos-Roubos *et al*. Frontiers in psychiatry 2022; 13 897138).

**Objectives:**

The aim of the case is providing a review of psychiartric and behavioral problems in PWS.

**Methods:**

Clinical case description and literature review on the subject.

**Results:**

We report a clinical case of a 23 year old man who was diagnosed with PWS. Clinical features includes intellectual disability, obesity, scoliosis bracing, probable hypoventilation-obesity syndrome [using non-invasive ventilation], hypercholesterolemia and hypogonadism. He took 3 doses of testosterone in 2017, which had to be suspended due to serious changes in behavior. Behavioral sporadic problems, reactive to the environment, are also present such as impulsiveness, stubbornness, aggressive outbursts, oppositional behavior, self-injuring behavior (placement of foreign bodies in the ear canal), card obsession and suspicious posture. This clinical condition has an impact on PWS relatives and at social level. He was medicated with Paliperidone 9mg; Topiramate 50mg; Clozapine 25mg**;** Escitalopram 10mg; and Haloperidol 2mg/ml (SOS). Currently, the patient is stable, with little weight gain and sporadic episodes of greater impulsivity without clinical relevance. He has participating in integrated activities at the institution.

**Conclusions:**

The main limitations in adolescence/adulthood are psychiatric and behavioral comorbidities, in association with hyperphagia and intellectual disability, which become more prominent with age. However, these symptoms are highly variable among individuals of different ages. Antipsychotics have been used for management of psychiatric and/or behavioral comorbidities. Other medications have also been used such as antidepressants (SSRI), antiepileptics, mood stabilizers and the response may vary depending on the individual. Weight gain, due to atypical antipsychotics, can be mitigated when food has controlled access. PWS has a major impact on the individual’s social and family environment, which requires an appropriate multidisciplinary strategy. A safe and constant environment as well as behavioral management programs must be ensured. (Butler *et al.* Current pediatric reviews 2019; 15 207-244).

**Disclosure of Interest:**

None Declared

